# Supplementary material for: Machine Learning Prediction and Phyloanatomic Modeling of Viral Neuroadaptive Signatures in the Macaque Model of HIV-Mediated Neuropathology
Source: Microbiol Spectr. 2023 Feb 27;11(2):e03086-22. doi: 10.1128/spectrum.03086-22 (PMC10100676; doi:10.1128/spectrum.03086-22)
Supplement: Supplemental file 1 — Supplemental material. Download spectrum.03086-22-s0001.pdf, PDF file, 0.4 MB [file spectrum.03086-22-s0001.pdf]

SUPPLEMENTAL MATERIAL

TABLE S1 Rules identified in PART model for classification of SIVE sequences. Nested rules, containing overlap of amino acid sites, were assigned a single prefix, resulting in six rule groups (1-6), ID = amino acid identity.

Rule	Feature	Value	Site
1_01	ID	W	344
1_01	ID	Q	216
1_01	ID	E	336
1_01	molecular size	> -3.656	62
1_02	ID	W	344
1_02	ID	E	336
1_02	molecular size	> -3.656	62
1_03	ID	W	344
1_03	ID	E	336
1_04	ID	W	344
1_04	ID	Q	216
1_04	molecular size	> -3.656	62
1_05	ID	W	344
1_05	ID	Q	216
1_06	ID	Q	216
1_06	molecular size	≤ -3.656	62
1_07	ID	K	336
2_01	ID	W	344
2_01	ID	P	420
2_01	ID	I	472
3_01	ID	P	420
3_01	ID	K	336
4_01	polarity	> -0.032	417
4_01	ID	Q	216
4_01	ID	I	472
4_02	polarity	> -0.032	417
4_02	ID	Q	216
4_03	polarity	> -0.032	417
4_03	ID	I	472
5_01	ID	W	344
5_01	ID	R	425
5_02	ID	R	425
6_01	ID	E	336
6_01	ID	I	472

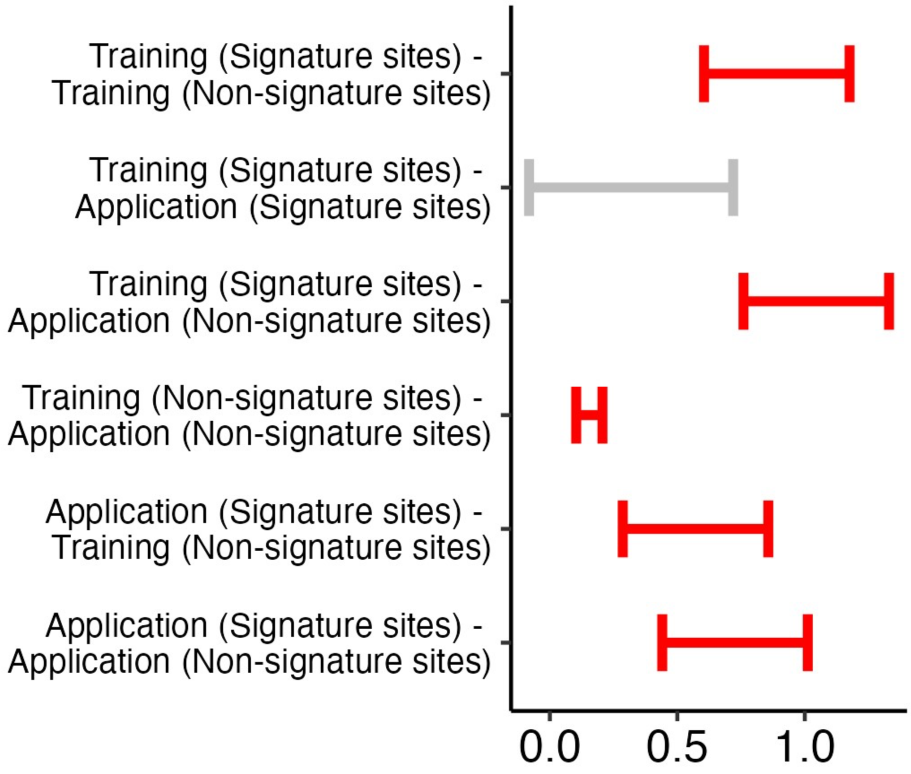

FIGS1 Mutationalentropydifferencesamongsignatureandnon-signaturesiteswithin training and application datasets. One-way ANOVA with Tukey's multiple comparisons *post hoc* test was performed in order to quantify relative differences in mutation entropy (x-axis) between classes (y-axis) and corresponding significance. *p–values* <0.01 were considered significant and indicated in red.

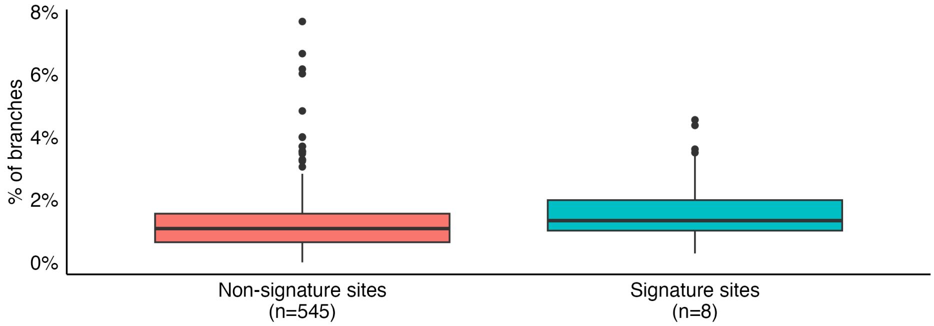

FIGS2 Pervasiveness of positive selection. Positive selection was identified for nucleotide sequence alignments from each animal using the mixed effects model of evolution (MEME)(32). The percentage of phylogenetic branches experiencing significant positive selection (y-axis) was determined for signature and non-signature sites (x-axis).

TABLE S2Detected signatures among animal models

Unit	Classification	SIVsm804E-CL757	SIVmac251	SIVmac251 + CD8+ depletion
Number of animals	TP[a]	3	1	3
	FP[b]	0	0	1
	TN[c]	0	2	0
	FN[d]	0	1	0
Number of sequences	TP[a]	113	31	104
	FP[b]	0	144	5
	TN[c]	0	6	27
	FN[d]	7	12	0

[a] True positives; [b] False positives; [c] True negatives; [d] False negatives

TABLE S3 HIV-1 and SIV envelope amino acid residues located in proximity to NAG residues in the superimposed PDB protein structure. Genbank reference based on the HIV-1 HXB2 accession K03455.1 (protein id AAB50262.1 and PDB 2NY3) and SIVmac251 accession KU892415.1 (protein id AMX21539.1 and PDB 3JCC). Bolded distances represent residues within the interaction distance threshold (4.5Å).

Envelope from	AA in PDB ref	Position in PDB ref	AA in Gen-Bank ref	Position in GenBank ref	Distance to NAG in angstroms (Å)
HIV-1	T	290	T	226	**3.9**
HIV-1	S	291	S	227	**3.6**
HIV-1	A	334	S	270	**3.5**
HIV-1	Q	341	Q	277	**4.0**
HIV-1	K	343	K	279	**3.0**
SIVmac251	W	332	G, Q, R, or W	344	**4.0**
SIVmac251	R	412	G, K, N, or R	425	**1.7**
SIVmac251	I	459	A, I, T, or V	472	10.7
